# Analysis of the roof damage range in close-proximity gently inclined coal seams mining and the feasibility of upward mining

**DOI:** 10.1038/s41598-025-89808-1

**Published:** 2025-02-13

**Authors:** Pei Zhang, Zhuo Li, Yibo Wei, Yang Chen, Liqiang Dong

**Affiliations:** College of Energy Engineering, Xi’an University of Science and Technology, Xi’an, 710054 China

**Keywords:** Close-proximity gently inclined coal seams, Upward mining, Depth of rock mass failure, Characteristics of the overlying rock collapsed, Stress transfer law of rock layer, Coal, Structural geology

## Abstract

In view of the feasibility of upward mining under the influence of repetitive mining for the close-proximity gently inclined coal seams, combined with the engineering geology of the coal seams in the south area of Xin’an Coal Mine, a formula for the depth of rock mass failure above the working face roof was proposed to investigate the continuity and integrity of coal seams 2^−3^ after the mining of the underlying coal seams. The characteristics of the overlying rock collapsed and the deformation law of the rock stratum sinking were analyzed through the similar experiments of physical simulation, to prove whether or not it is technically feasible to mine upward for the coal seams. Numerical simulation software is used to simulate the spatial distribution of mining stress field and stress transfer law of rock layer in the process of coal seams mining. The study shows that coal 2^−3^ is located within the lower coal seam fissure zone. The rock layer at the bottom of the working face has a certain bearing capacity, and can still maintain good continuity under the influence of repetitive mining. The stress concentration area of coal 3 up-slope mining develops continuously to the upper left rock body, and the peak of stress concentration is getting closer and closer to the coal wall, and the stress of coal 2^−3^ bottom plate and coal 2^−3^ top plate does not fall back significantly after the peak of stress occurs. The degree of rock fall and damage after mining is small, meeting the conditions required for upward mining. The results of the study provide a reference for the analysis of overburden structure and feasibility assessment under similar coal seams upward mining conditions.

## Introduction

In recent years, as high-quality coal resources with better endowment conditions have been gradually extracted, many mines are facing the problem of re-mining of upland legacy coal resources, and how to improve the comprehensive utilization of underground space and legacy resources is the main challenge of safe and efficient mining of coal resources^1–4^. Under some special conditions, upstream mining can rapidly increase the production capacity of mines and the construction speed of new wells, reduce the amount of roadway works and maintenance, and alleviate the contradiction of mining area succession.

Scholars have conducted a large number of studies on upward mining of coal seams and achieved many valuable results. Sun et al.^5^ used theoretical analysis to derive the discriminative formula of “partially balanced structure formation level” and “balanced structure formation level” in upward mining, and proposed a discriminative steps for whether the upper coal seam can be mined under the condition of large-height integrated mining open space; Yuan et al.^6^ constructed the parameter of mining thickness of the lower coal seam, spacing of coal seams and lithology index of interbedded rock strata as the parameters, and put forward a method for discriminating the feasibility of upward mining of close coal seams based on the statistical analysis of the data; Chunlei^7^ analyzed the upward mining of overburden rock collapse and transport law of the group of near-distance seams with the group of near-distance seams as the research background; Cui et al.^8^ established a key layer structural mechanics model for the inverted trapezoidal overburden structure of the upper part of the coal seam of strong impact tendency with the impact occurring in a critical position, and proposed a key layer structural mechanics model of the coal seam. Critical layer structural mechanics model, put forward the analysis method of the minimum safety distance of the coal pillar; Shao et al.^9^ studied the overburden cleavage evolution law and the stability of the interlayer rock layer in the process of upward mining of a typical face of the East Northwest Coal Mine; Wang et al.^10^ studied the structural morphology of the top and bottom plates under the repetitive mining action of upward mining, and proposed the “three-hinged arch structure of upward mining”, as well as derived the stability condition criteria and calculation equations of the model. Li et al.^11^ studied the typical characteristics and formation mechanism of the caving roof morphology in the goaf, and proposed the concept of the “positive-inverted triangle” caving roof morphology in the goaf. Sun et al.^12^ have shown that the effective bearing time of coal pillars in goaf is closely related to the rheological properties of coal pillars. By establishing a mechanical model and analyzing influencing factors, they have provided a calculation formula and analysis method for the stability of coal pillars in goaf. Feng et al.^13^ analyzed the evolution law of abutment pressure during the up-going longwall mining process in the residual mining area of the knife-pillar through similar simulation experiments, and established a stress model for the advanced mining pillars based on the key stratum theory. Feng et al.^14^ proposed a theory and method for determining the technical conditions for up-going fully mechanized mining in residual mining areas using the caving method. By analyzing the interlayer rock control layers and deformation basins, they provided a feasible determination for up-going mining in abandoned coal seams. Feng et al.^15^ studied the movement and deformation of interlayer rock strata in the overlying coal seam of the mined-out area through similar simulation experiments, revealing the existence of interlayer rock strata control layers and the characteristics of rock strata movement and deformation. Feng et al.^16^ studied the dynamic stability of the remaining pillars under the influence of up-going mining, revealing the impact of up-going disturbance loads on the stability of the remaining pillars, providing a theoretical basis for the safe mining of pillar-type goaf. Wu et al.^17^ analyzed the impact of interlayer rock on upward mining under special conditions, established a mechanical model of “suspended structure”, and verified the stability of the structure during the upward mining process through numerical simulation and similar simulation. Yang et al.^18^ studied the impact of the mining sequence of multiple coal seams under the river on the development height of the water-conducting fissure zone in the overlying rock strata. Through theoretical analysis and numerical simulation methods, they determined the height of the water-conducting fissure zone under different mining sequences and proposed a reasonable mining sequence. Hu et al.^19^ studied the development law of overburden fractures in the process of mixed mining of coal seams, and found that the degree of fracture development is affected by the composite thickness, interlayer spacing, and mining sequence of coal seams. They established a logarithmic relationship model between the ratio of fracture mining and the composite thickness of coal seams. Zhao et al.^20^ proposed a cross-pillar roadway layout scheme for the problem of roadway layout in the uphill mining face of close-spaced coal seams, by analyzing the impact and damage degree of mining on the overlying coal seams. Zhang et al.^21^ studied the characteristics of rock failure, fracture evolution, and subsidence deformation during the uphill mining process in deep coal seams, and analyzed the relationship between the subsidence deformation curve of the rock strata and the development of fractures and fissures, as well as the stress state. Yang et al.^22^ discussed the mining design and engineering application of improving the upper limit of mining under the condition of kicking the air. Through theoretical calculations and FLAC 3D numerical simulation tests, they analyzed the characteristics of overburden movement and abutment pressure in mining with and without kicking the air. Wu et al.^23^ analyzed the hazards in the process of up-going mining, combined with the results of on-site microseismic monitoring, divided the recovery process of the working face into different stages, identified the maximum hazards, and took corresponding safety measures. Wang et al.^24,25^ proposed an improved particle swarm optimization (PSO) algorithm to calibrate the microscopic parameters of the discrete element method (DEM) model and, through the simulation (SA-GEP) method, established a new empirical formula for uniaxial compressive strength (UCS) based on Schmidt hammer rebound value. Wang et al.^26,27^ compared the performance of CEM, DE, EFO, MFO, and SSO algorithms in calibrating the DEM model’s microscopic parameters, determined the optimal hyperparameters, and discussed the mechanical and fracture characteristics of rock samples with rough, non-persistent joints. Wang et al.^28–31^ explored the peak strength, fusion, and failure process of rock materials with two pre-existing cracks and those containing preset joints and circular holes through experimental and numerical studies. They also experimentally investigated crack propagation and fusion in rock materials with two pre-existing cracks under biaxial compression. Mangal^32^ reviewed conventional methods for analyzing roof collapse mechanisms and support resistance requirements in longwall mining and examined the roof collapse mechanisms in longwall working faces. Guo et al.^33^ assessed the stability of goaf areas based on a combination of weight and cloud model, and validated the accuracy of the model using FLAC3D numerical simulations. Mangal^34^ described the stratigraphic mechanics and convergence monitoring during pillar removal in thick coal seams using the anchor cable method in single-lift mining. Bai et al.^35^ introduced a new method for evaluating the chain instability of remaining coal pillars in up-going mining, based on the method of removing components. They applied this method to evaluate the risk of chain instability of remaining coal pillars under the influence of upward mining. Yixin et al.^36^ conducted research on the stress and fracture evolution laws of surrounding rock under the condition of short-distance coal seam mining, and determined the stability and fracture development morphology of the floor rock strata of the working face. Yong et al.^37^ explored the feasibility of up-going mining technology under the condition of thick coal seam caving, and analyzed the characteristics of the development of overburden rock damage zones. Cao et al.^38^ studied and analyzed the feasibility of uphill mining in the gob-side entry retaining section of the “three-soft” thin coal seam group. Fu et al. introduced a water prevention technique for uphill mining in close-spaced coal seams. By analyzing the causes of water filling in the goaf and the characteristics of water accumulation, they propose a water drainage technique under pressure to prevent outbursts. Dai et al. proposed a method by inversely-inclined slicing and upward mining to reduce ground subsidence and deformation in ultra-thick coal seams^39^. Liu et al. validated the feasibility of multi-seam upward mining in Shanxi’s Dianping coal mine, enhancing resource recovery and waste-free mining^40^. Various methods were used to analyze the deformation and damage characteristics, fissure development law and mining stress of the coal seam overburden under repetitive mining^41–44^. In addition, the critical filling rate and critical height of filling up-going mining were also studied^45,46^.

This paper takes Xin’an Coal Mine as the background, studies the integrity of the coal 2^−3^ floor rock strata under the influence of repeated mining in closely spaced gently dipping coal seams using theoretical analysis, physical similarity simulation experiments, and numerical simulation methods. On this basis, a reasonable upward mining sequence for the coal seam groups in the southern area is determined.

## Engineering background

**Fig. 1:**
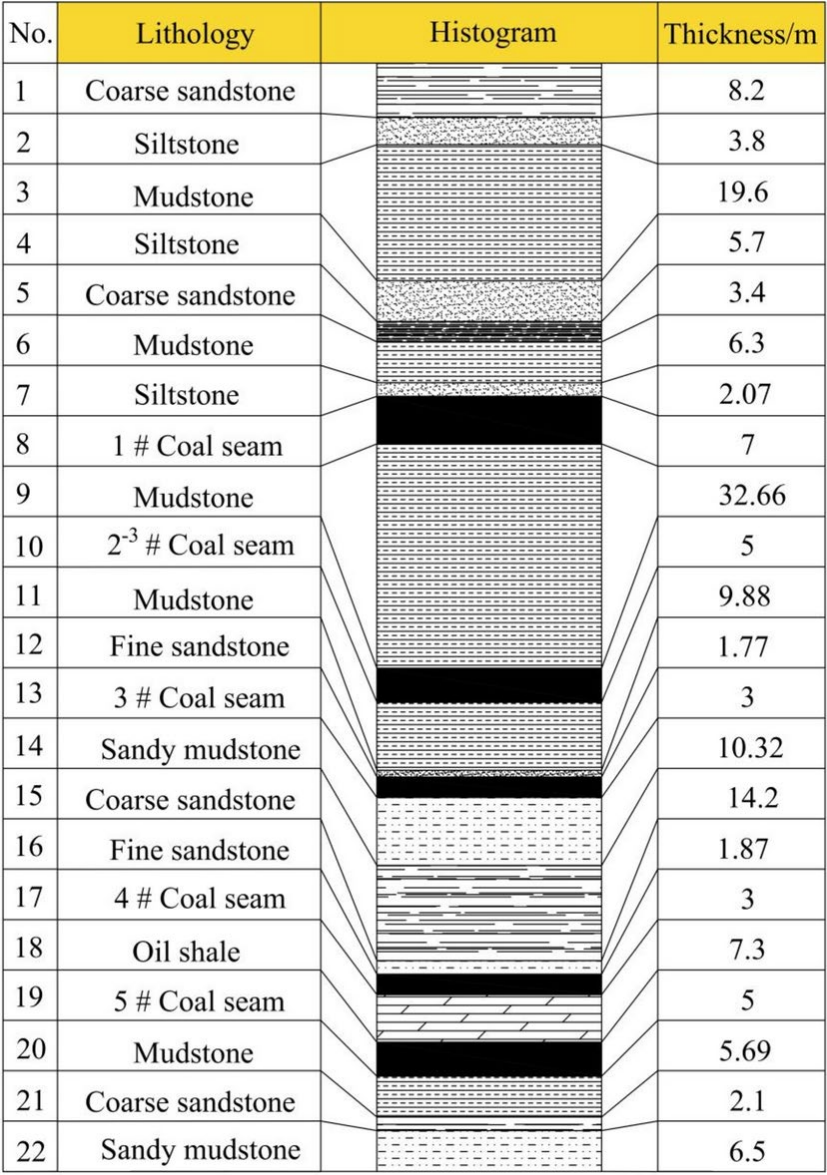
Strata diagram.

The Xin’an Coal Mine is located in Xinyao Town, Chongxin County, Pingliang City, Gansu Province. The mine is approximately 3.8 km long from north to south and 0.6–1.6 km wide from east to west, covering an area of about 4.513 km^2^. The mine field is divided into two mining areas by an elevation of + 535 m, with the main roadway of the mining area serving as the boundary between the northern and southern areas. There are a total of five mineable coal seams in the mine, from top to bottom: Coal 1, Coal 2^−3^, Coal 3, Coal 4, and Coal 5. The southern section of the near-horizontal mining area for Coal 1 is divided into the working faces 1201, 1203, 1205, and 1207. Extraction of Coal 1 in this area has been completed. In the southern section of the near-horizontal mining area, Coal 3 is divided into the working faces 3203, 3205, and 3207, with the mining sequence following 3205 → 3203 → 3207. The 3205 working face has been fully mined. The 3203 working face has a minable strike length of 1980 m, a face length of 233 m. The 3207 working face has a minable strike length of 2050 m, a face length of 180 m. The next phase of mining will focus on gradually completing the extraction of Coal 3. The extraction of Coal 3 is expected to induce damage to the overlying strata, which will inevitably affect the overlying Coal 2–3 seams, thus raising concerns regarding the upward mining of the coal seam group. Coal seam 2^−3^ has a thickness ranging from 1.69 to 5.93 m, with an average thickness of 5 m and an average dip angle of 9°, and is on average 32.66 m away from Coal 1. Coal seam 3 has a thickness ranging from 1.2 to 7.1 m, with an average thickness of 3 m and an average dip angle of 9°, and is on average 11.65 m away from coal seam 2^−3^. The geological histogram is shown in Fig. 1.

## ‘Three-zone’ identification method

**Fig. 2:**
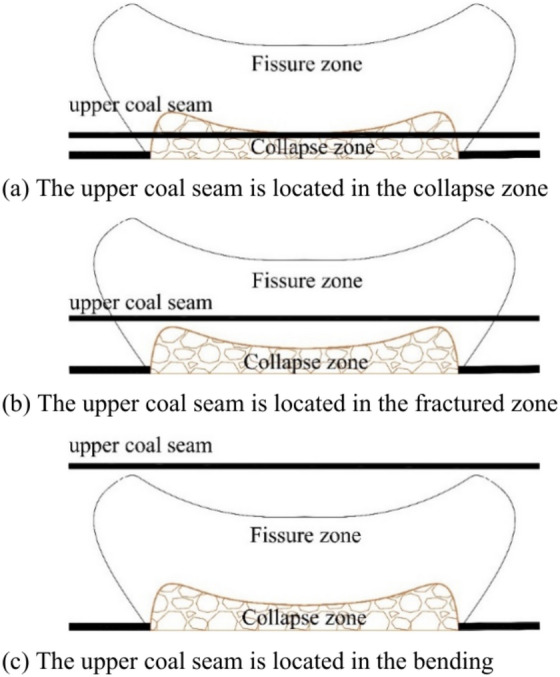
The ‘three-zone’ identification method.

When using the longwall full-caving mining method, after the movement of the overlying strata in the goaf stabilizes, from bottom to top, a collapse zone, a fissure zone, and a bending subsidence zone will form. According to the ‘three-zone’ identification method, after the lower coal seam is mined, if the upper coal seam is within its collapse zone, upward mining of the upper coal seam is not feasible. If the upper coal seam is within the fissure zone, mining can be carried out with certain measures. If the upper coal seam is within the bending subsidence zone, upward mining of the upper coal seam is feasible. The ‘three-zone’ identification method for upward mining is shown in Fig. 2.

According to the research of Cheng et al.^47^, the empirical formula of the development height of caving zone and fracture zone is obtained:

1 $$H_{k} = \frac{100\sum M }{{4.7\sum M + 19}} \pm 2.2$$

2 $$H_{li} = \frac{100\sum M }{{1.6\sum M + 3.6}} \pm 5.6$$

In the formula, H_k_ is the height of collapse zone, H_li_ is the height of fissure zone, and M is the mining thickness of coal seam.

Based on the data from borehole K2 in the southern area, an analysis was conducted on the impact of mining coal seam 3 on coal seam 2^−3^. The thickness of coal seam 3 is approximately 3 m, and the average distance between coal seam 3 and coal seam 2^−3^ is 11.76 m. After the mining of coal seam 3, the height of the caving zone ranges from 6.86 to 11.26 m, with the highest point reaching the bottom edge of coal seam 2^−3^. Coal seam 2^–3^ is primarily located within the fracture zone of the overlying strata of coal seam 3, maintaining an overall stratified continuity.

After the completion of mining coal seam 3 in the southern area, coal seam 4 is mined. The caving zone height of coal seam 4 is between 6.86 and 11.26 m, and the height of the fracture zone ranges from 30.1 to 41.3 m. The distance between coal seam 4 and coal seam 3 is 26 m, indicating that the caving zone of coal seam 4 does not penetrate into coal seam 3. Consequently, the development height of the caving zone in coal seam 3 after the mining of coal seam 4 remains consistent with that observed during the isolated mining of coal seam 3. Therefore, the mining of coal seam 4 after the completion of coal seam 3 will not cause any structural damage to coal seam 2^−3^.

The distance between coal seam 5 and coal seam 4 is 6.71 m, and the caving zone height of coal seam 5 ranges from 6.44 to 9.44 m, placing coal seam 4 within the caving zone of coal seam 5. Therefore, the plan to mine coal seam 5 first followed by the upward mining of coal seam 4 is not feasible. According to the “three-zone” identification method, after the mining of coal seams 3 and 4, the caving zone height of coal seam 5 ranges from 6.44 to 9.44 m, while the fracture zone height ranges from 37.5 to 48.7 m.

When the caving zone of the lower coal seam contacts or fully penetrates the upper coal seam, the maximum height of the fracture zone in the upper seam should be calculated based on the thickness of the upper seam. Meanwhile, the maximum height of the fracture zone in the lower seam should be calculated based on the combined mining thickness of both coal seams. The combined mining thickness M_z1–2_ of the upper and lower coal seams is as follows:

3 $$M_{z1 - 2} = M_{2} + \left( {M_{1} - \frac{{h_{1 - 2} }}{{y_{2} }}} \right)$$

In the formula, M_1_ is the thickness of the upper coal seam; M_2_ is the thickness of the lower coal seam; h_1–2_ is the normal distance between M_1_ and M_2_; y_2_ is the ratio of the collapse height of the lower coal seam to the mining height.

$$M_{z1 - 2} = M_{2} + \left( {M_{1} - \frac{{h_{1{-} 2} }}{{y_{2} }}} \right) = 5 + \left( {3 - \frac{7}{9.44/5}} \right) = 4.29\,{\text{m}}$$

$$H_{li} = \frac{100\sum M }{{1.6\sum M + 3.6}} \pm 5.6 = \frac{100 \times 4.29}{{1.6 \times 4.29 + 3.6}} \pm 5.6 = 41 \pm 5.6\,{\text{m}}$$

The development height of the fracture zone for the mining of coal seams 4 and 5 is 41 ± 5.6 m. The distance between coal seam 4 and coal seam 2^−3^ is 40 m, indicating that the caving zone has not extended into coal seam 2^−3^, while the fracture zone has reached coal seam 2^−3^. After the downward mining sequence of coal seams 3, 4, and 5 in the southern area, the caving zone has not developed into coal seam 2^−3^, which maintains its overall stratified continuity. With appropriate measures, coal seam 2^−3^ can be mined.

## Analysis of the scope of roof damage

### Stress distribution in the vicinity of the working face of the lower coal seam mining

**Fig. 3:**
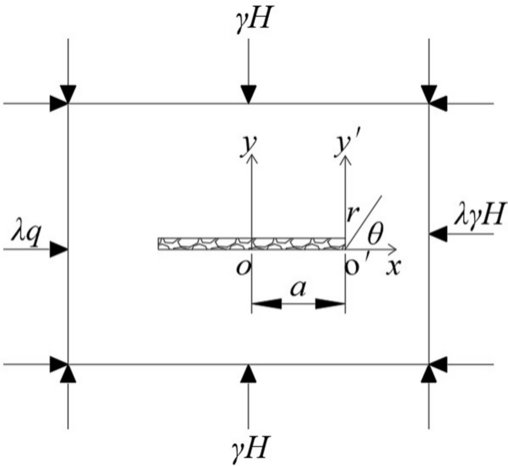
The simplified stress diagram of the surrounding rock.

The extraction of the lower coal seams leads to changes in the mechanical environment of the surrounding rock during the mining of the upper coal seams, which in turn induces deformation of the surrounding rock and may further lead to its failure. When mining coal seams using the longwall caving method, due to the significantly greater strike length of the working face compared to the mining height, the stress environment around the mining area can be simplified to the mechanical model shown in Fig. 3.

Let the tendency length of mining *L* = 2*a*, the stress distribution in the vertical direction is *γH* (*γ* is the average capacity weight of the overlying rock layer on the working face; *H* is the burial depth of the coal seam), and the stress distribution in the horizontal direction is *λγH* (*λ* is the horizontal stress coefficient). At the determined point (*r*, *θ*), As the mining length *L* of the working face increases, the stress around the working face becomes greater, and the degree of concentration in the stress concentration zone increases. In practical calculations $r < < L$, *λ* is usually taken as 1. Therefore, the stress distribution around the working face can be rewritten as:

4 $$\left\{ {\begin{array}{*{20}l} {\sigma_{x} = \frac{\lambda H}{2}\sqrt{\frac{L}{r}} \cos \frac{\theta }{2}\left( {1 - \sin \frac{\theta }{2}\sin \frac{3\theta }{2}} \right)} \hfill \\ {\sigma_{x} = = \frac{\lambda H}{2}\sqrt{\frac{L}{r}} \cos \frac{\theta }{2}\left( {1 + \sin \frac{\theta }{2}\sin \frac{3\theta }{2}} \right)} \hfill \\ {\tau_{xy} = \frac{\lambda H}{2}\sqrt{\frac{L}{r}} \cos \frac{\theta }{2}{\text{in}}\frac{\theta }{2}\sin \frac{3\theta }{2}} \hfill \\ \end{array} } \right.$$

According to the theory of elasticity, the stress distribution in the vicinity of the working face is brought to the principal stress formula can be obtained:

5 $$\left\{ {\begin{array}{*{20}l} {\sigma_{1} = \frac{\lambda H}{2}\sqrt{\frac{L}{r}} \cos \frac{\theta }{2}\left( {1 + \sin \frac{\theta }{2}} \right)} \hfill \\ {\sigma_{2} = \frac{\lambda H}{2}\sqrt{\frac{L}{r}} \cos \frac{\theta }{2}\left( {1 - \sin \frac{\theta }{2}} \right)} \hfill \\ {\sigma_{3} = 0\;\left( {Plane\;stress} \right)} \hfill \\ {\sigma_{3} = \frac{\mu \lambda H}{2}\sqrt{\frac{L}{r}} \cos \frac{\theta }{2}\;\left( {Plane\;strain} \right)} \hfill \\ \end{array} } \right.$$

In the formula: *μ* is the Poisson’s ratio of the surrounding rock near the working face.

### Calculation of the extent of damage in the vicinity of the working face

#### Analysis of the failure zone near the working face under plane stress conditions

Assuming that the surrounding rock near the mining area follows the Mohr–Coulomb criterion during failure, then:

6 $$\sigma_{1} - \xi \sigma_{1} = R_{c}$$

In the formula: $\xi = \frac{1 + \sin \varphi }{{1 - \sin \varphi }}$; *φ* is the internal friction angle of the rock mass; *R*_*c*_ is the uniaxial compressive strength of the rock mass.

Substituting formula (5) into the Mohr–Coulomb criterion formula, the boundary formula of the failure zone at the edge of the working face under plane stress conditions is obtained as:

7 $$r = \frac{{\gamma^{2} H^{2} L}}{{4R_{c}^{2} }}\cos^{2} \frac{\theta }{2}\left( {1 + \sin \frac{\theta }{2}} \right)^{2}$$

When *θ* = 0, the length *r*_0_ of the failure zone in the horizontal direction at the edge of the working face can be obtained from the above formula as $r_{0} = \frac{{\gamma^{2} H^{2} L}}{{4R_{c}^{2} }}$.

**Fig. 4:**
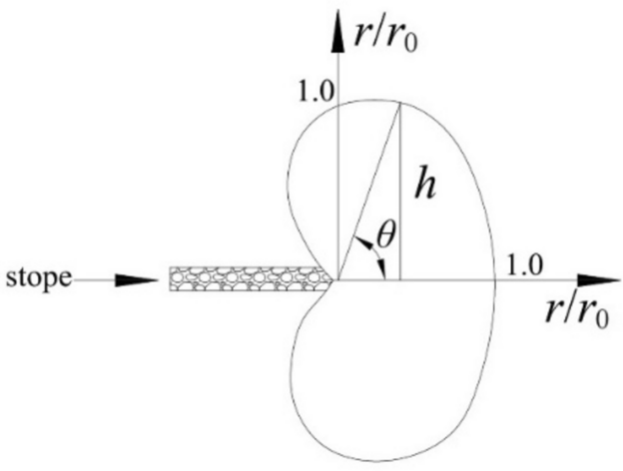
Diagram of rock mass failure near the working face.

From formula (7), the shape of the failure zone at the edge of the working face caused by stress concentration can be obtained, as shown in Fig. 4.

The depth of the rock mass failure in the roof above the edge of the working face h can be calculated as:

8 $$h = \frac{{\gamma^{2} H^{2} L}}{{4R_{c}^{2} }}\cos^{2} \frac{\theta }{2}\left( {1 + \sin \frac{\theta }{2}} \right)^{2} \sin \theta$$

By setting $\frac{dh}{{d\theta }} = 0$, the maximum failure depth *h*_*max*_ of the roof rock mass can be obtained. By differentiating formula (8) and simplifying, it can be concluded that when $\sin \frac{\theta }{2} = \frac{1 + \sqrt 7 }{6}$, h can obtain a maximum value of *h*_*max*_ as:

9 $$h_{\max } = \frac{{1.57\gamma^{2} H^{2} L}}{{4R_{c}^{2} }}$$

The horizontal distance *L*_*m*_ from the maximum failure depth of the roof rock mass to the end of the working face is:

10 $$L_{m} = h_{\max } \cot \theta = \frac{{0.42\gamma^{2} H^{2} L}}{{4R_{c} }}$$

#### Calculation of the failure zone near the working face under plane strain conditions

Substituting formula (5) into formula (6), the boundary formula of the failure zone near the working face under plane strain conditions is obtained as:

11 $$r^{\prime} = \frac{{\gamma^{2} H^{2} L}}{{4R_{c}^{2} }}\cos^{2} \frac{\theta }{2}\left( {1 + \sin \frac{\theta }{2} - 2\xi \mu } \right)^{2}$$

When *θ* = 0, the length *r*_0_′ of the failure zone in the horizontal direction at the edge of the working face under plane strain conditions can be obtained from the above formula as:

12 $$r_{0} ^{\prime} = \frac{{\gamma^{2} H^{2} L\left( {1 - 2\xi \mu } \right)^{2} }}{{4R_{c}^{2} }}$$

The failure depth *h*_0_′ of the rock mass above the roof of the working face under plane strain conditions is:

13 $$h_{0} ^{\prime} = \frac{{\gamma^{2} H^{2} L}}{{4R_{c}^{2} }}\cos^{2} \frac{\theta }{2}\left( {1 + \sin \frac{\theta }{2} - 2\xi \mu } \right)^{2} \sin \theta$$

In the formula, *λ* is the unit weight of the rock mass; *H* is the burial depth of the coal seam; *L* is the mining length of the working face; *R*_*c*_ is the uniaxial compressive strength of the rock mass.

Comparing formulas (8) and (13), it can be seen that the failure zone at the edge of the working face under plane stress conditions is larger than that under plane strain conditions. The above calculations do not consider the rock mass in the yield failure zone, which undergoes stress yielding and plastic flow. If this condition were taken into account, the extent of the failure zone would increase further. The derivation of formula (9) only considers the lateral scale of the goaf, without taking into account its vertical scale, which corresponds to the roof failure height of the working face. In reality, the goaf has a vertical height, which should include the mining height of the lower coal seam *M* and the caving zone height *H*_*m*_ of the underlying rock layers. After considering the vertical scale of the goaf, the maximum failure height of the roof above the working face can be rewritten as:

14 $$h_{\sigma } = \frac{{1.57\gamma^{2} \left( {H - M - H_{m} } \right)^{2} L}}{{4R_{c}^{2} }} + H_{m}$$

By combining the formula analysis with the mining of coal seam 3 at Xin’an Coal Mine, the following parameters are given: Working face mining height = 3.0 m; Coal seam burial depth = 412 m; Caving zone height of the underlying rock layers = 6 m; Working face mining length = 180 m; Uniaxial compressive strength of the rock mass = 39.6 MPa; Unit weight of the rock mass = 23.5 kN/m^3^. Substituting these parameters into formula (13), the maximum failure height of the roof above the working face is calculated to be 10.04 m. After mining the lower coal seam, the maximum failure height of the roof is 10.04 m. The average distance between coal seam 3 and coal seams 2^−3^ is 11.76 m. Since the maximum failure height does not affect coal seams 2^−3^, coal seam 2^−3^ can be mined normally to a certain extent.

## Physical similarity simulation experiment

### Model building

Based on the geological columnar section, as well as the occurrence, lithology, and mechanical parameters of the roof and floor, the similar material ratio parameters are selected according to similarity theory, and the material ratio for the similarity model of the southern area is designed, as shown in Table 1 (The selected screened river sand is used as aggregate, plaster and lime powder are employed as binding materials. In the proportion number, the first digit represents the proportion of river sand in the filling material, whereas the second and third digits indicate the proportional relationship between the two binding materials within the binding agent).

**Table 1:** Material ratio for the similarity model of the southern area.

No.	Lithology	Thickness of rock layer/m	Thickness of model/cm	Proportion number	Material consumption/(kg/cm): amount per layer			
					River sand	Plaster	Lime powder	Coal ash
1	Siltstone	32	16	828	8.75	0.22	0.86	–
2	Coarse sandstone	8	4	837	8.64	0.32	0.76	–
3	Siltstone	8	4	828	8.75	0.22	0.86	–
4	Mudstone	44	22	937	8.75	0.29	0.68	–
5	Coarse sandstone	24	12	837	8.64	0.32	0.76	–
6	Fine sandstone	6	3	928	8.75	0.19	0.78	–
7	Mudstone	40	20	937	8.75	0.29	0.68	–
8	Coarse sandstone	8	4	837	8.64	0.32	0.76	–
9	Siltstone	4	2	828	8.75	0.22	0.86	–
10	Mudstone	20	10	937	8.75	0.29	0.68	–
11	Siltstone	6	3	828	8.75	0.22	0.86	–
12	Coarse sandstone	3	1.5	837	8.64	0.32	0.76	–
13	Mudstone	6	3	937	8.75	0.29	0.68	–
14	Siltstone	2	1	937	8.75	0.29	0.68	–
15	1 # Coal seam	7	3.5	20:1:5:20	3.43	0.17	0.86	3.43
16	Mudstone	33	16.5	928	8.75	0.19	0.78	–
17	2–3 # Coal seam	5	2.5	20:1:5:20	3.43	0.17	0.86	3.43
18	Mudstone	10	5	937	8.75	0.29	0.68	–
19	Fine sandstone	1	0.5	928	8.75	0.19	0.78	–
20	3 # Coal seam	3	1.5	20:1:5:20	3.43	0.17	0.86	3.43
21	Sandy mudstone	10	5	928	8.75	0.19	0.78	–
22	Coarse sandstone	14	7	837	8.64	0.32	0.76	–
23	Sandy mudstone	2	1	928	8.75	0.19	0.78	–
24	4 # Coal seam	3	1.5	20:1:5:20	3.43	0.17	0.86	3.43
25	Oil shale	7	3.5	919	8.75	0.1	0.9	–
26	5 # Coal seam	5	2.5	20:1:5:20	3.43	0.17	0.86	3.43
27	Mudstone	6	3	928	8.75	0.19	0.78	–
28	Coarse sandstone	2	1	837	8.64	0.32	0.76	–
29	Sandy mudstone	6	3	828	8.75	0.22	0.86	–

**Fig. 5:**
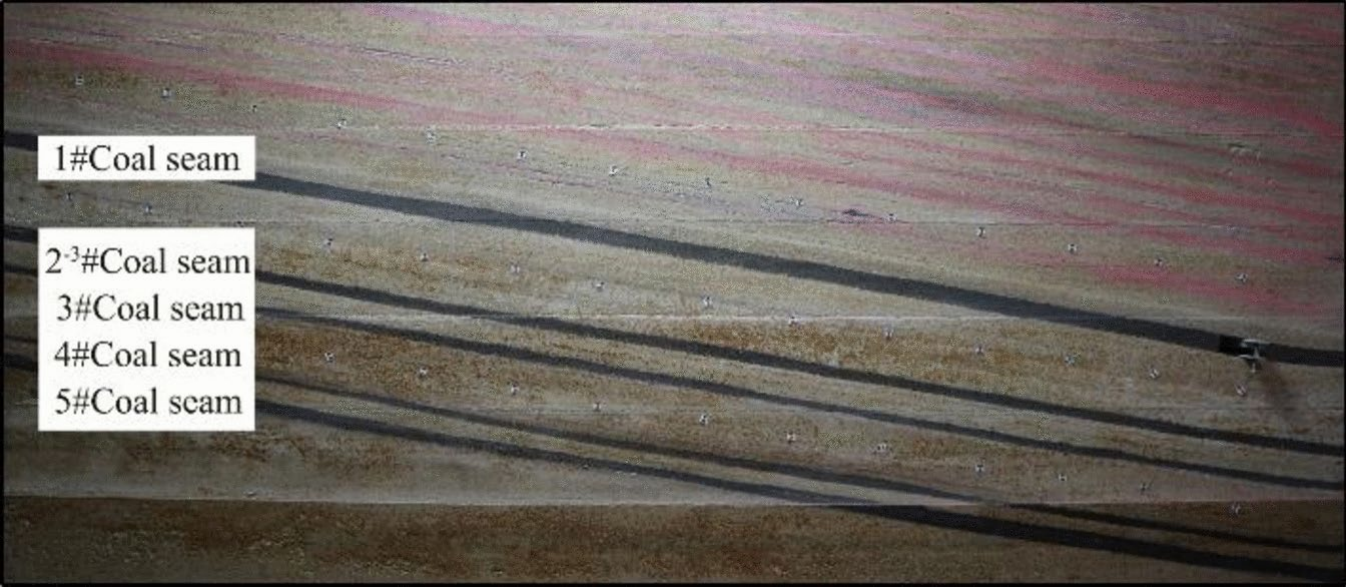
Physical similarity simulation model.

The model experiment framework for mining the gently inclined coal seam group in the southern area is designed with a uniform dip angle of 9° based on the average dip angle of coal seam 5. The advancing length along the strike of the working face in the southern area is about 2000 m. A plane model frame of 300 cm × 20 cm × 200 cm is used for the physical similarity material model experiment (Fig. 5). The geometric similarity ratio of the model (prototype: model) is 200, the time similarity ratio is 14.14, the density similarity ratio is 1.5625, the stress similarity ratio is 312.5.

### Measurement point layout and mining design

**Fig. 6:**
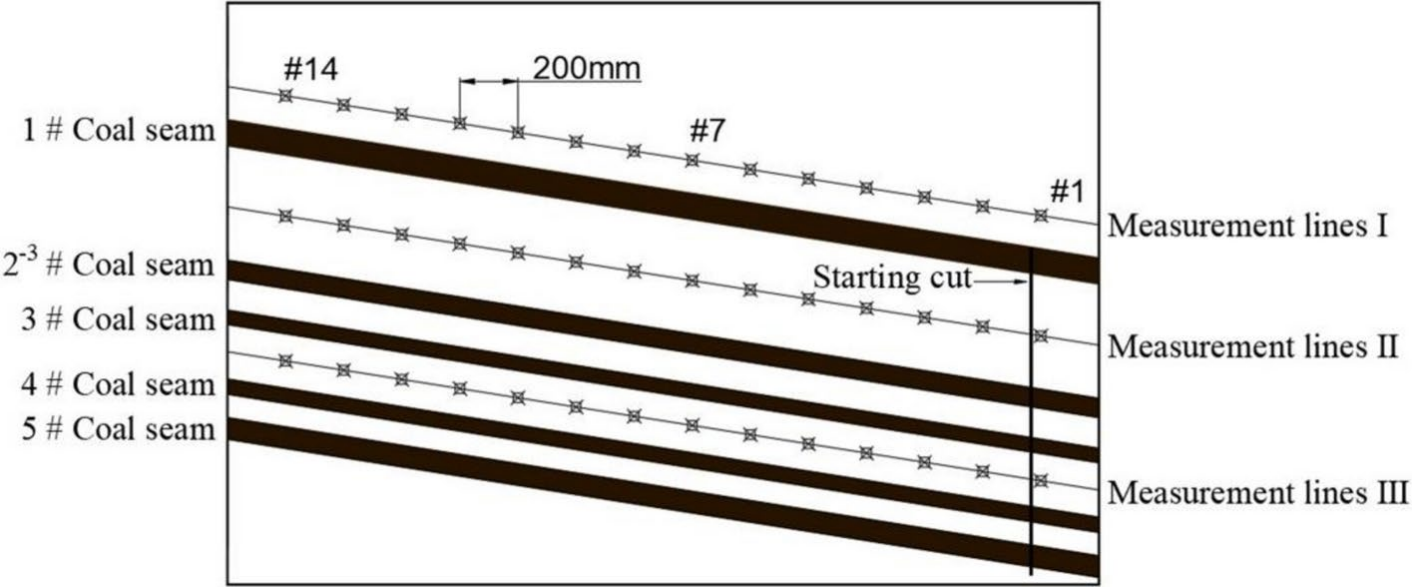
Displacement measurement point arrangement.

To study the migration and variation patterns of coal seam 2^−3^ under the repeated mining influence conditions of coal seam 3, coal seam 4, and coal seam 5, three rows of displacement measurement lines were arranged on the model. Each row consists of 14 displacement measurement points to observe the displacement changes in the coal seams, with a horizontal spacing of 200 mm between measurement points, as shown in Fig. 6.

To eliminate boundary effects and ensure the accuracy of the experimental conclusions, a boundary of 200 mm is left around the nearly horizontal mining area of the southern model (40 m in actual engineering). According to the incline length of the mining area and the mining design, the actual incline length of the working face is determined to be 280 m, with each coal layer’s mining time in the model calculated to be approximately 7 min using the time similarity ratio. Based on actual mining practices, the southern working face progresses with upward mining (from south to north, from low to high). First, the upper coal seam, coal seam 1, is mined to simulate real mining conditions, and after the overburden is stabilized, the lower coal seam group is then mined.

### Overburden collapse and its migration law in coal seam mining

**Fig. 7:**
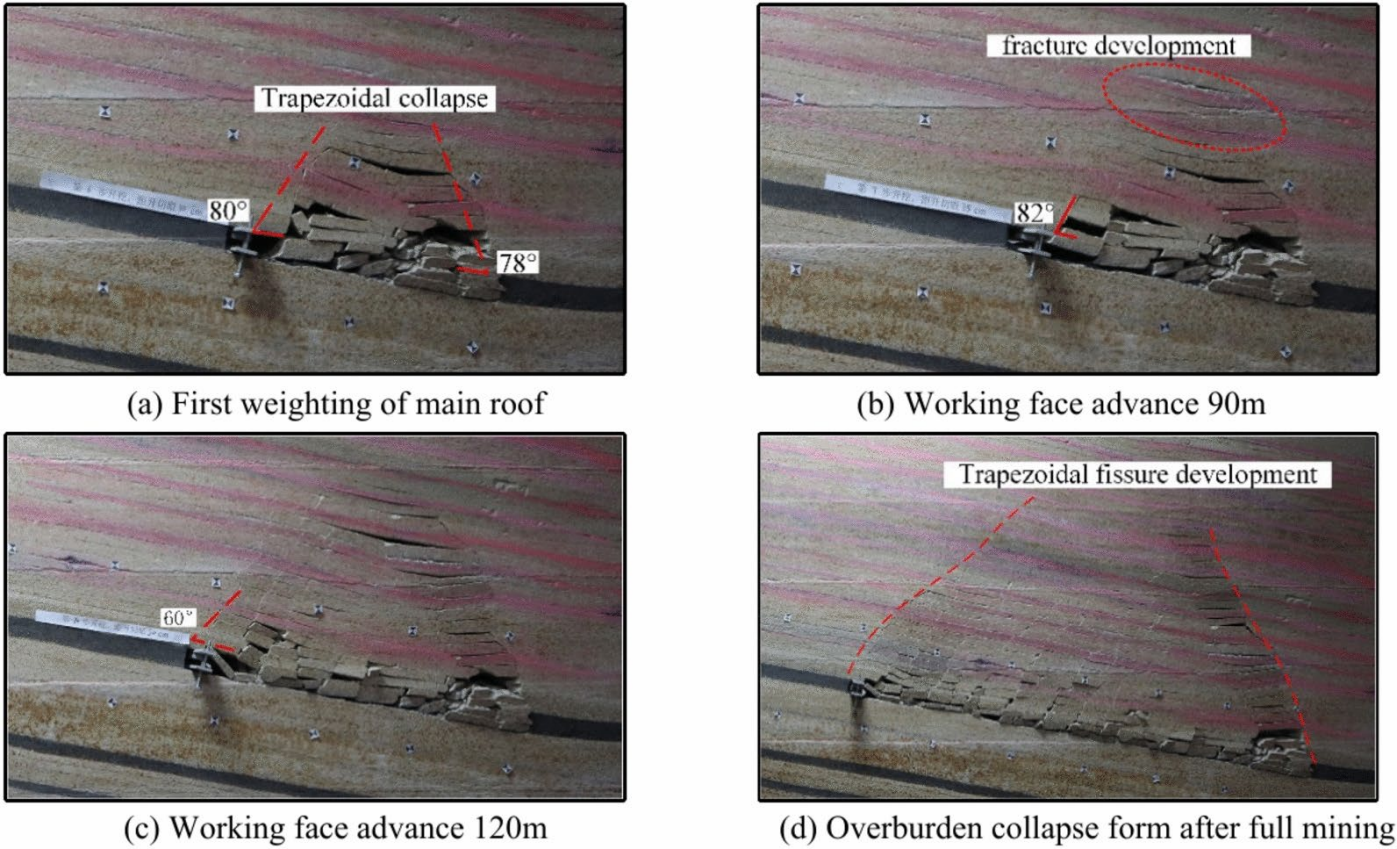
Characteristics of structural damage to coal 1 roof and its overlying rock strata.

The average mining height of coal seam 1 is 7 m, with a 33 m thick mudstone layer separating it from coal seams 2^−3^. The model adopts an inclined mining method from right to left. When the working face advances to 80 m, the immediate roof experiences its initial weighting, and the overburden exhibits a “trapezoidal” collapse with a collapse height of 48 m. The collapse angle on the cut-off side is 78°, and the collapse angle on the advancing side is 80°, as shown in Fig. 7a.

When the working face advances to 90 m, the immediate roof undergoes its first periodic weighting with a weighting interval of 10 m. The collapse height is 22 m, and the collapse angle on the advancing side is 82°, as shown in Fig. 7b. When the working face advances to 120 m, fractures further develop, and the collapse angle on the advancing side changes to 60°, as shown in Fig. 7c.

When the working face advances to 230 m, the immediate roof undergoes its 15th periodic weighting, with a weighting interval of 10 m, and the development height of the separation layer reaches 182 m. After this, the development height of the separation layer remains unchanged, indicating that the working face has reached full mining extent at 230 m, with a total mining distance of 230 m, as shown in Fig. 7d.

**Fig. 8:**
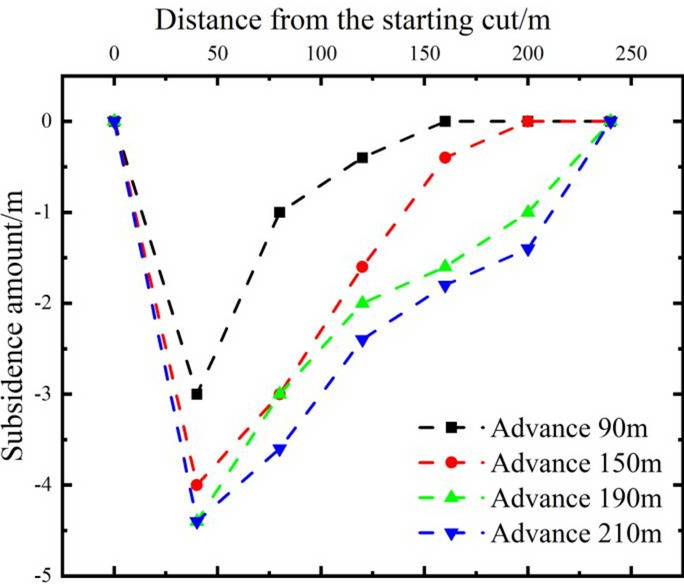
Characteristics of the displacement distribution of the A measuring line after coal 1 is mined back.

The vertical displacement curves of survey line I after mining coal seam 1 at different advancing distances show that coal seam 1 has reached full mining extent, as shown in Fig. 8. The measured height of the collapse zone is about 28 m, and the height of the fissure zone is about 182 m.

**Fig. 9:**
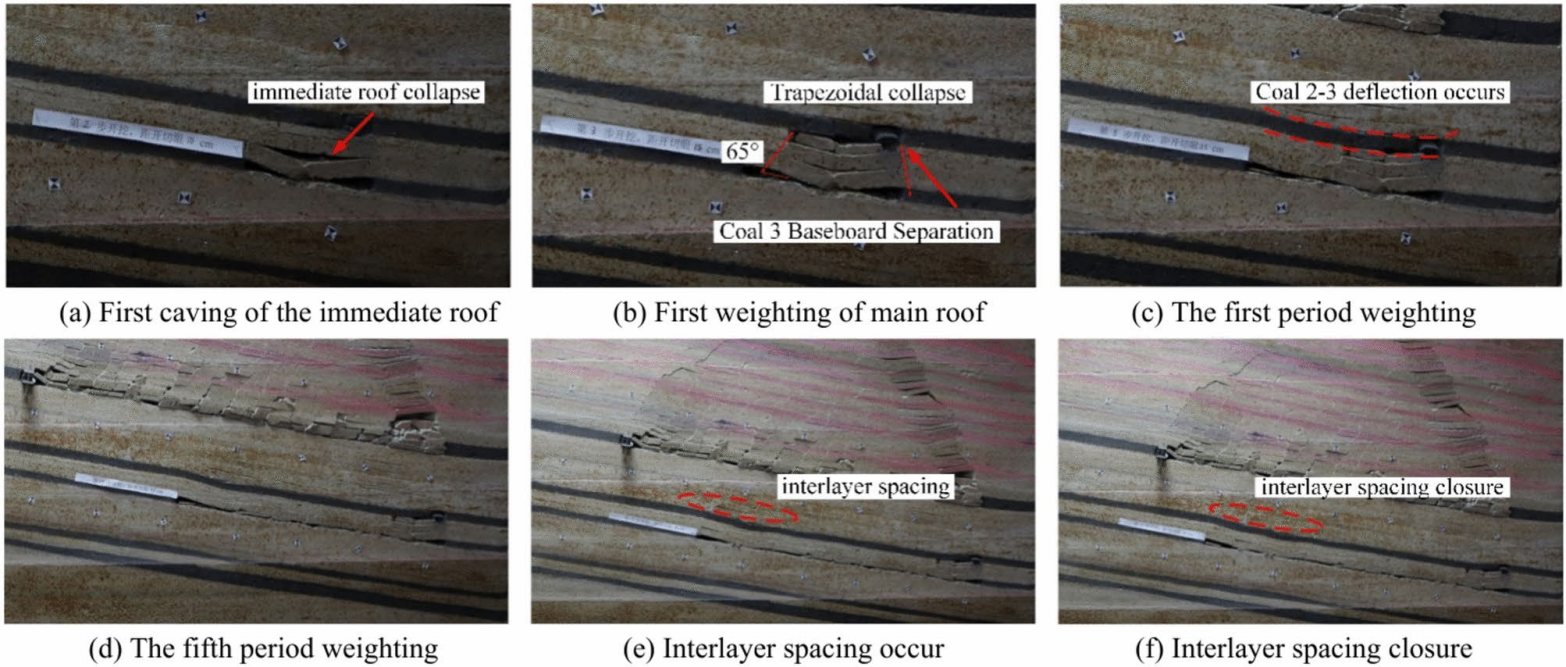
Characteristics of structural damage to coal 3 roof and its overlying rock strata.

The interlayer between coal seam 3 and coal seam 2^−3^ consists of 1 m thick fine sandstone and 10 m thick mudstone. When the working face of coal seam 3 is mined to 40 m, the immediate roof of coal seam 3 collapses, and the collapse height is 3.5 m, as shown in Fig. 9a. When the working face of coal seam 3 advances to 50 m, the main roof undergoes its first weighting, and the overlying strata exhibit a “trapezoidal” collapse, with a separation layer appearing at the floor of coal seam 2^−3^, as shown in Fig. 9b. When the working face of coal seam 3 advances to 70 m, the first periodic weighting of the main roof occurs, with a weighting step of 20 m, and flexure appears in the lower part of coal seam 2^−3^, as shown in Fig. 9c. When the working face of coal seam 3 advances to 80 m, the separation layer is significantly developed, with a separation height of 34.6 m. A total mining distance of 240 m was achieved, and nine periodic weightings of the main roof were recorded. The final collapse pattern of the overlying strata after the mining of coal seam 3 is shown in Fig. 9d.

Observations from the weighting cycles indicate that there are both large and small periodic weightings in coal seam 3. As the working face advances, the separation in the overlying strata continues to develop. The immediate roof experiences a progression from noticeable separation development to compaction of the separation and closure of fractures, as shown in Fig. 9e and f. The caving angle on the side of the cut-off trench is 75°, while the caving angle on the advancing side is about 65°–85°, with an average of about 73°. The mining of coal seam 3 has a minor impact on coal seam 2^−3^, resulting in some flexure while maintaining an overall layered continuous structure. The full mining distance is 240 m. The measured fissure zone of coal seam 3 is 49.8 m, with a fracture-to-mining ratio of 16.6. After the extraction of coal seam 3 is completed, the overlying coal seam 2^−3^ remains in a stratified, overall continuous state, within the fissure zone caused by the mining of coal seam 3. This indicates the feasibility of upward mining in coal seam 2^−3^.

**Fig. 10:**
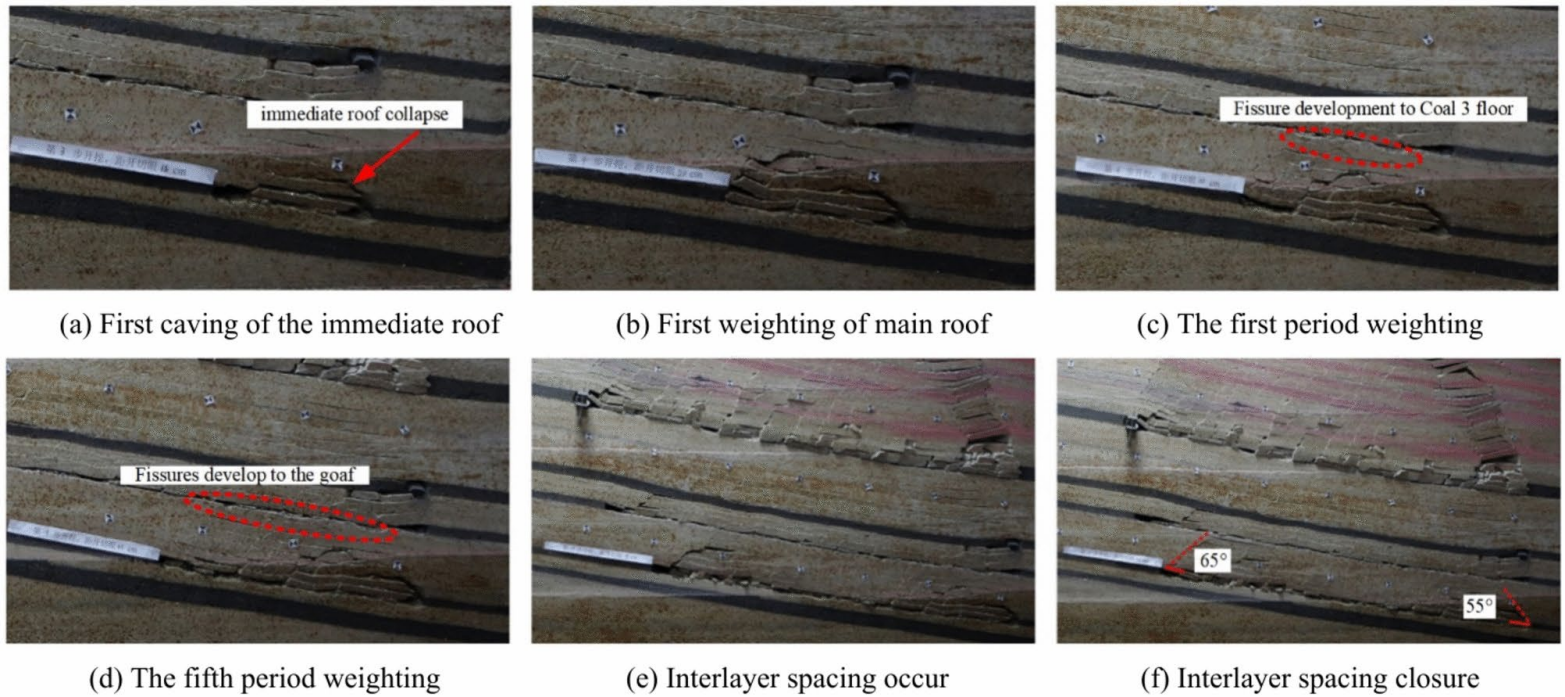
Characteristics of structural damage to coal 4 roof and its overlying rock strata.

When the coal seam 4 working face was mined for 30 m, the immediate roof of coal seam 4 collapsed, and the collapse height was 4.2 m, as shown in Fig. 10a. When the coal seam 4 working face was advanced to 40 m, the main roof was pressed for the first time, and the overburden collapsed in a “trapezoidal” manner, with a collapse height of 12.6 m, a collapse angle of 55° on the starting cut, and a collapse angle of 70° on the advancing side, as shown in Fig. 10b. When the coal seam 4 working face was advanced to 50 m, the roof was pressed for the first time, with a weighting step of 10 m, and the stratum developed to the bottom of coal 3, as shown in Fig. 10c. When the coal seam 4 working face advances to 150 m, the fifth periodic compression of the old roof causes continued development of the separation zone, and cracks extend to the coal seam 3 goaf, as shown in Fig. 10d. When the coal seam 4 working face was advanced to 190 m, the eighth periodic weighting of the main roof occurs, with a weighting step of 15 m, as shown in Fig. 10e. The total mining distance was 230 m, recording nine periodic weighting of the main roof. The periodic weighting step distance for coal seam 4 is 20 m. The collapse angle on the starting cut was 55°, and the collapse angle on the advancing side was 65°–80°. The fractures in coal seam 4 extend to the goaf of coal seam 3. The distance between coal seam 4 and coal seam 3 is 26.39 m, and the collapse zone of coal seam 4 has not caused a significant impact on the goaf of coal seam 3. The final overlying strata collapse pattern is shown in Fig. 10f.

**Fig. 11:**
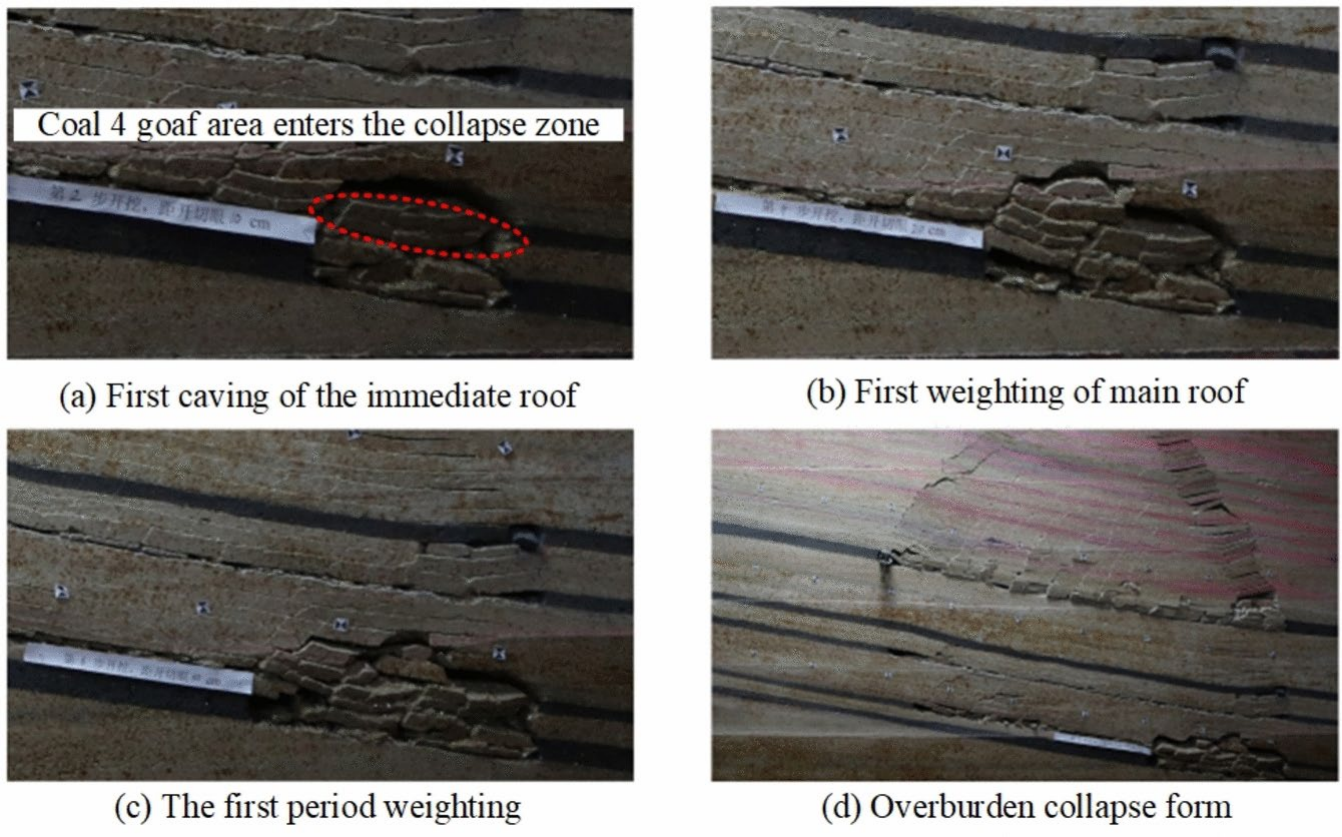
Characteristics of structural damage to coal 5 roof and its overlying rock strata.

When the coal 5 working face was mined for 30 m, the immediate roof collapses with a collapse height of 4.8 m. Between coal seam 5 and coal seam 4, there is a 3.5 m oil shale, and the coal seam 4 goaf directly enters the collapse zone, as shown in Fig. 11a. Since there was 3.5 m oil shale above coal 5 and the coal seam 4 goaf above, the roof within a certain range above was a broken roof, and the interval rock layer collapsed as it was mined. During the mining process, the pressure was not obvious, but only a periodic collapse phenomenon was presented, as shown in Fig. 11b and c. Since the thickness of the interval layer between coal 5 and coal 2^−3^ was large, the mining of coal 5 had little impact on coal 2^−3^, and the overall layered continuous structure of coal 2^−3^ remained intact. The final collapse pattern of the overlying strata after the mining of coal seam 5 is shown in Fig. 11d.

The characteristics of overburden collapse during the mining of various coal seams are summarized in Table 2. During the processes of initial weighting and periodic weighting, changes in the collapse angle significantly influence the stability of the roof strata. The collapse angle directly affects the height of the collapse zone and the stability of the roof. A larger collapse angle leads to an increase in the height of the collapse zone, reducing the support area of the roof strata, which makes the occurrence of initial and periodic weighting more likely. As mining progresses downward through the coal seam group, the collapse angle of each seam gradually decreases, and the height of the collapse zone shows a declining trend. During the mining of the upper coal seams, roof fractures become more concentrated and developed, with the height of fracture development also decreasing in tandem with the reduction in the height of the collapse zone. The step distance of periodic weighting correspondingly decreases. Coal seam 1 and its roof undergo continuous damage during the mining of the lower coal seams, and the collapse zone of coal seam 1 becomes more fragmented compared to the collapse zones of the lower seams after experiencing damage and the effects of three mining cycles.

**Table 2:** Overburden caving characteristics of each coal seam mining.

Coal seam	Initial weighting/m	Cyclic weighting/m	Straddle angle of starting cut/°	Straddle angle of propulsion side (Average)/°	Rockfall height/m	Fissure development height/m
Coal seam 1	80	10	78	80	28	182
Coal seam 3	50	20	75	73	11	49.8
Coal seam 4	40	20	55	75	12.6	29
Coal seam 5	30	15	60	68	4.8	–

Throughout the experiment, similarities are observed between the evolution of rock structure and the development of fractures. Before the direct roof collapses, fractures dynamically develop and propagate from the mining entry toward the goaf at angles ranging from 60° to 90°. The fractures in the roof of the coal seam undergo stages of initiation, extension, divergence, and eventual penetration, leading to the collapse of the direct roof. Due to the bulking effect, the collapse zone and the overlying fractured zone form a load-bearing structure. As the rock strata stabilize, the separation space is gradually compressed, and the height of fracture development no longer changes. The rock strata in the stable region of the fractured zone can maintain their integrity.

After the mining of coal seam 1 is completed, the downward mining of coal seams 3, 4, and 5 continues. The overlying remnant coal seam 2^−3^ remains in a continuous, stratified, and intact state, indicating the feasibility of mining coal seam 2^−3^.

## Simulation analysis of overburden under repeated mining activities

To further analyze the characteristics of overburden damage during multi-seam mining, numerical simulation software was used to complement the limitations of similar material simulation experiments. Through numerical simulations, the stress distribution, overburden damage characteristics, and their spatiotemporal evolution during the mining process of coal seam groups were further analyzed.

### Numerical model construction

**Fig. 12:**
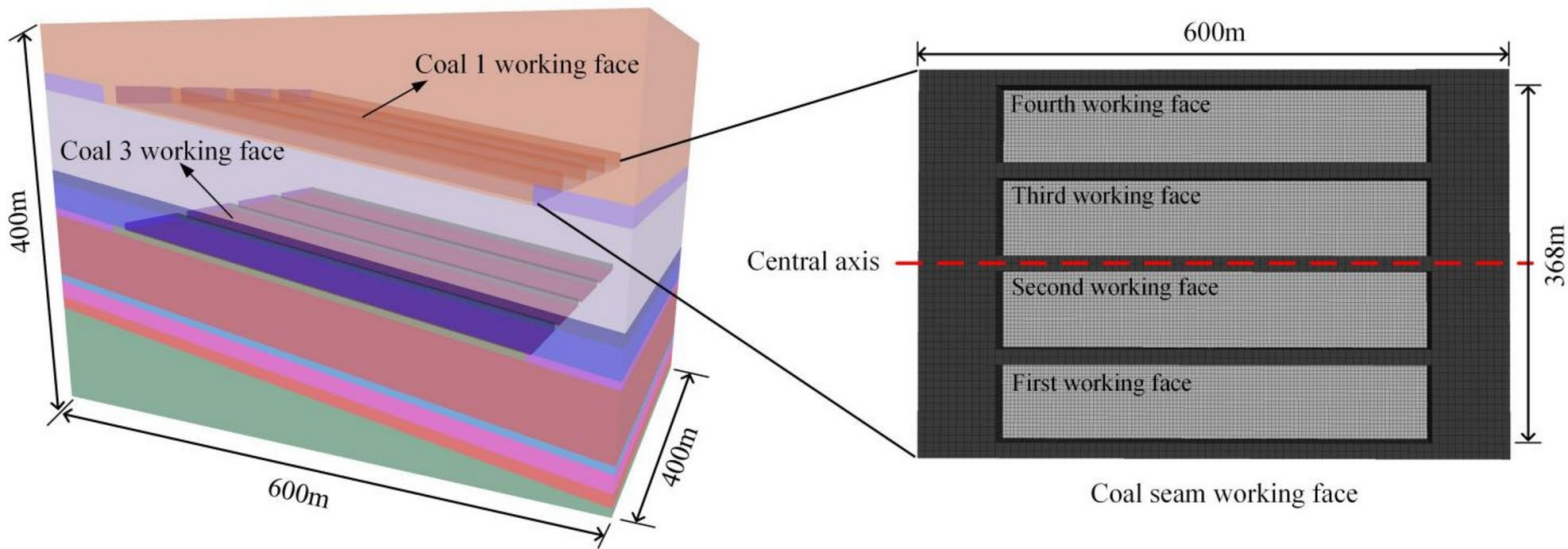
Model building.

According to the actual mining plan of the coal mine, a three-dimensional numerical model was established using FLAC3D. The model dimensions are as follows: strike length (x-direction) of 600 m, dip length (y-direction) of 400 m, and height (z-direction) of 400 m, as shown in Fig. 12. The coal and rock strata are simulated using the ideal Mohr–Coulomb plastic yield criterion. Based on rock mechanics experiments and coal seam column diagrams, the physical and mechanical parameters of each layer in the model are provided in Table 2. The left, right, and bottom boundaries of the model are fixed displacement constraint boundaries, while the upper boundary is a stress boundary. To account for boundary effects, the mining boundary on the left side is 80 m away from the left boundary of the model, and the mining boundary on the right side is 80 m away from the right boundary of the model. A pressure of 16.6 MPa is applied to the top layer of the overburden, and the working face advancement uses inclined mining. The mining sequence is as follows: coal seam 1 → coal seam 3 → coal seam 4. The simulation analyzes the stress distribution and damage evolution characteristics of the overburden at different advancement distances for the working faces of coal seams 3 and 4.

### Initial mining-induced stress field spatial distribution

**Fig. 13:**
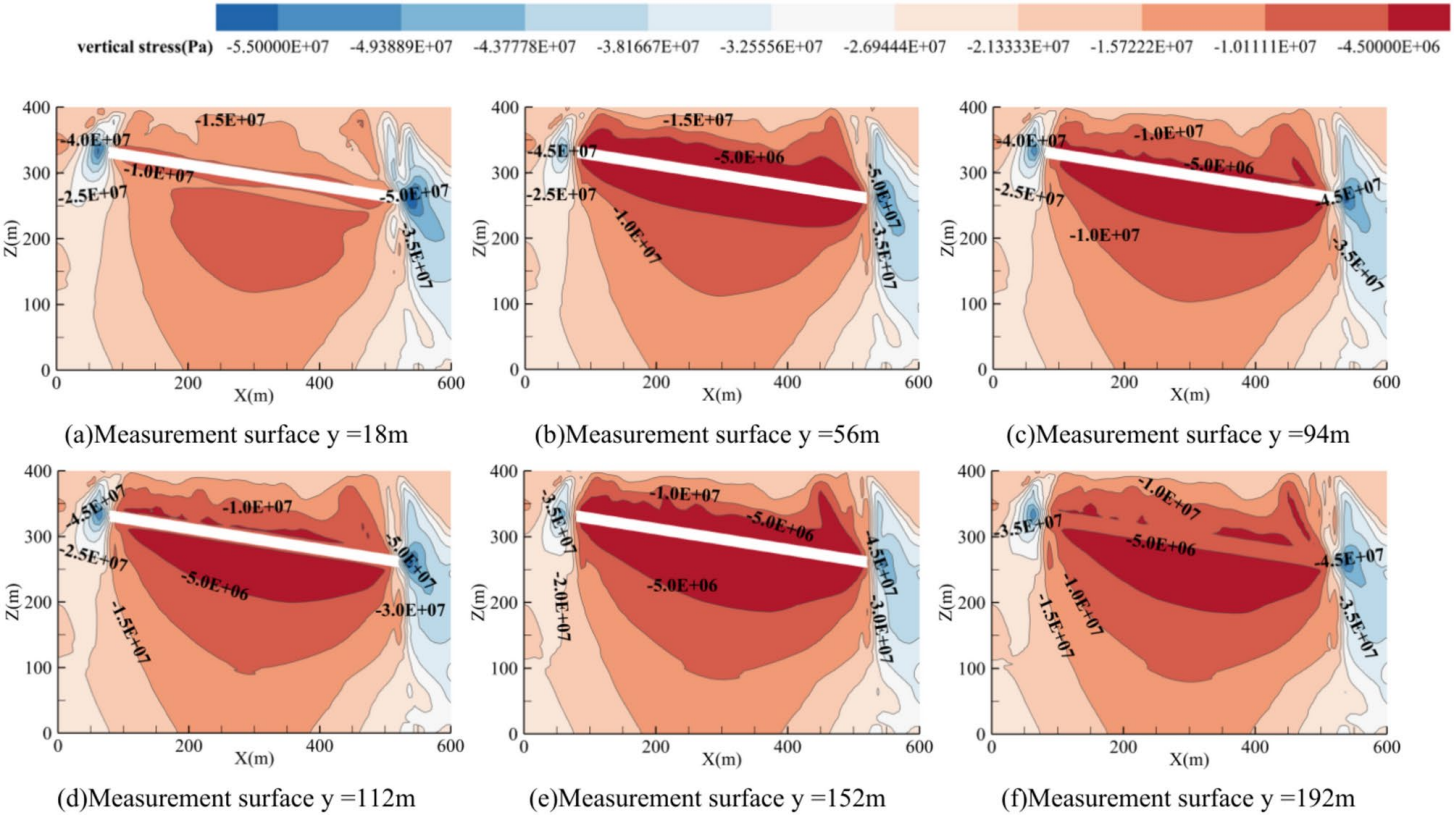
The dip cloud chart after the mining of the first working face of coal 1.

Vertical stress plays a critical role in coal seam mining, directly affecting the stability of the roof and the safety of the mine, especially during the mining of closely spaced coal seams. Therefore, after the completion of coal seam 1 mining, stress variations along the dip and strike directions were analyzed by placing cross-sections perpendicular to the y-axis and x-axis. In the dip direction, the rock strata near the open cut and coal wall areas form vertical stress concentration zones (with asymmetric distribution). Stress concentration occurs near the roof at the open cut, and a critical stress arch forms in the roof of the goaf, indicating a stress release zone. The stress distribution characteristics of the surrounding rock are shown in Fig. 13.

**Fig. 14:**
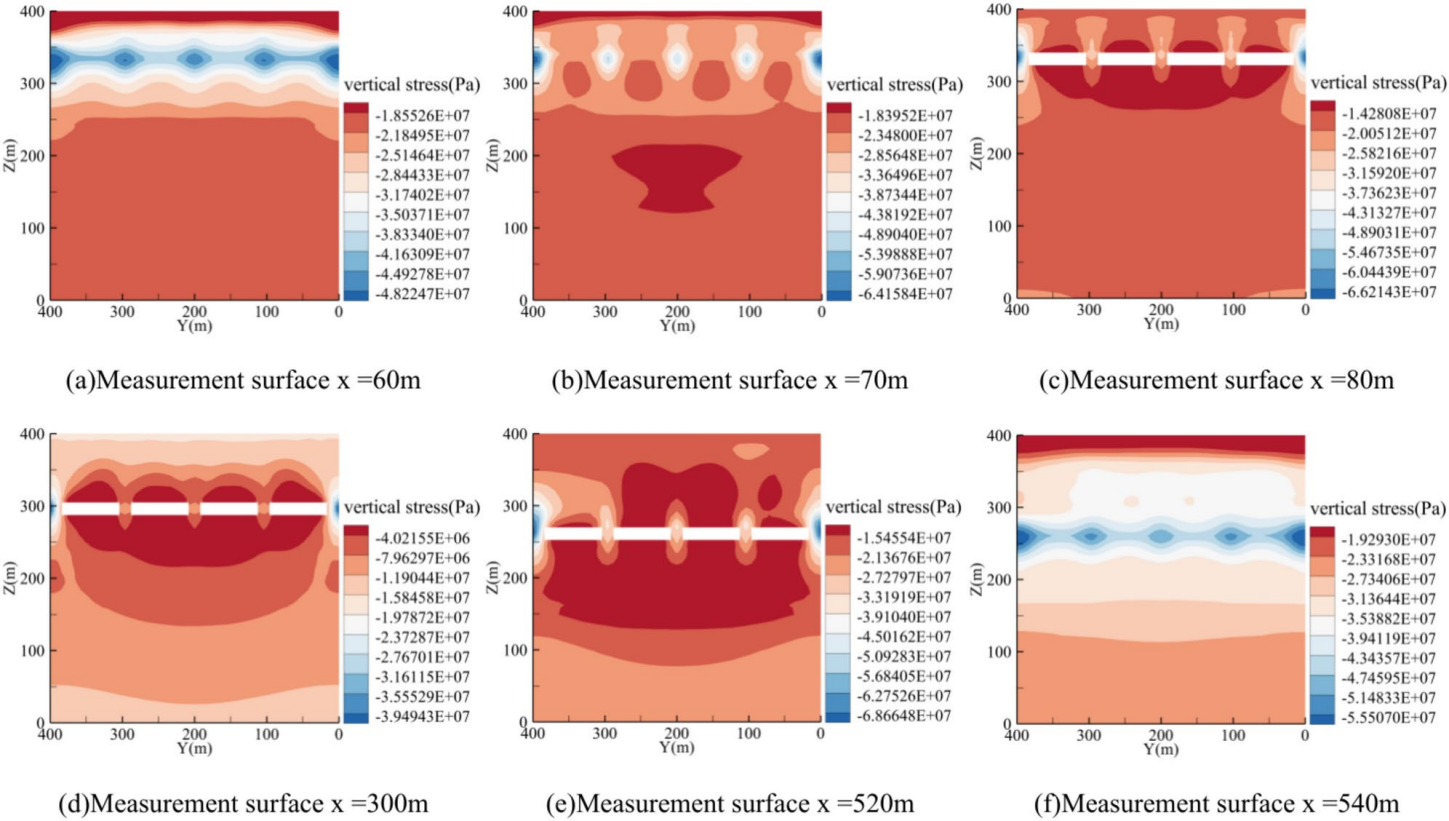
The strike cloud chart after the mining of the first working face of coal 1.

After the initial mining of the working face, the vertical stress in the stope exhibits regional characteristics. Along the dip direction of the coal seam, the stope stress can be divided into the original rock stress zone, stress concentration zone, pressure relief zone, and stress redistribution zone. After the upper coal seam is mined, the stress in the surrounding rock layers of the recovery space is redistributed. This not only causes stress concentration in the coal pillars around the recovery space but also transfers this stress to the deeper layers of the floor rock. In the strike direction, vertical stress concentrates at the coal pillars and the two side boundaries, with the stress distribution showing a symmetrical pattern. The stress concentration in the central part of the goaf is lower than on both sides of the coal wall (Fig. 14).

### The evolution characteristics of surrounding rock stress

After the mining of the upper coal seam, the stress in the surrounding rock layers of the recovery space is redistributed, leading to severe roof fracturing and intense pressure when mining the lower coal seam. In multi-seam mining, stress concentration zones and pressure relief zones also exist. During the mining of the lower coal seam (coal seam 3), the advancing abutment pressure from the working face, combined with the stress concentration caused by the mining of coal seam 1, results in a high-stress superposition effect. This can easily cause rock bursts or other mining disasters, and appropriate measures should be taken to prevent such hazards. Compared to single-seam mining, the stress concentration coefficient in multi-seam mining increases, and the stress evolution in multi-seam mining exhibits a superposition effect.

**Fig. 15:**
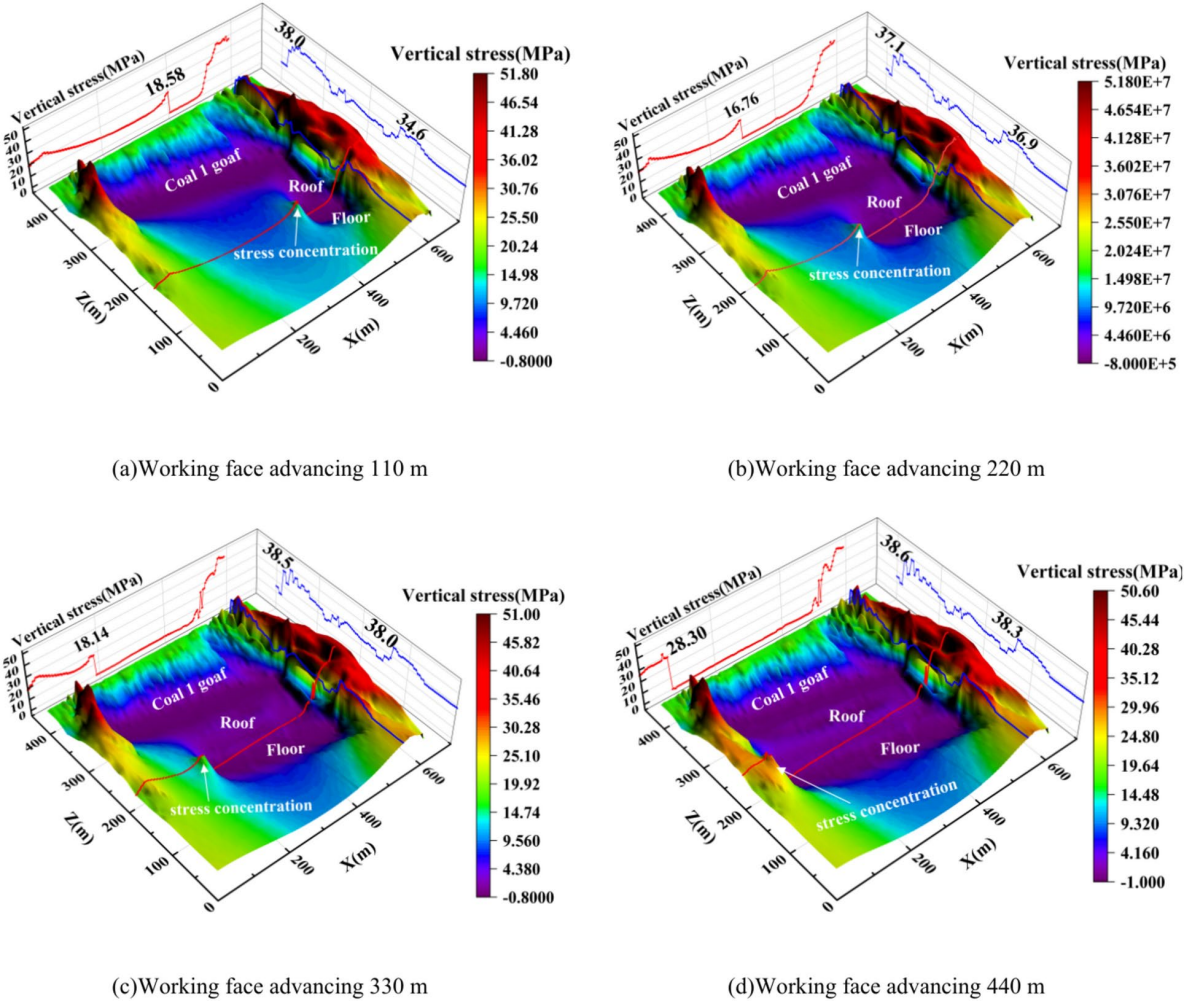
Characterization of spatial stress field distribution in coal 3 mining.

After the mining of coal seam 1, based on the actual mining conditions, the first working face of the lower coal seam (coal seam 3) was mined in sections. After completing each section, a cross-section was arranged along the center of the goaf (y = 56 m) to extract surrounding rock stress data at different mining stages and analyze the evolution characteristics of the surrounding rock stress. Figure 11 shows the cloud maps of vertical stress after each section is mined. After the first section was mined, the peak vertical stress in front of the advancing coal wall was located at x = 400.8 m, z = 145 m, with a magnitude of 18.58 MPa (Fig. 15a). After the second section was mined, the stress concentration area shifted upward, and the peak vertical stress in front of the advancing coal wall was at x = 293.7 m, z = 160 m, with a magnitude of 16.76 MPa, a decrease of 9.8% compared to the first section (Fig. 15b). After the third section was mined, the goaf area increased, and the pressure relief zone between coal seams expanded accordingly, with greater stress release from the roof. The peak vertical stress in front of the advancing coal wall was at x = 186.5 m, z = 180 m, with a magnitude of 18.15 MPa, an 8.3% increase compared to the second section but still less than the peak stress of the first section (Fig. 15c). After the fourth section was mined, the high-stress zone in front of the coal wall overlapped with the stress concentration zone of the upper coal seam, reaching a peak vertical stress of 28.30 MPa at x = 75.4 m, z = 200 m (Fig. 15d).

As the mining advances along the inclined direction, the degree of stress concentration in front of the coal wall at the open cut continues to increase. At 530 m from the open cut in coal seam 3, as each section is mined, the stress levels are 34.6 MPa, 36.9 MPa, 38.0 MPa, and 38.3 MPa, respectively. The distance between the peak stress concentration in front of the coal wall and the coal wall itself decreases progressively across the four mining segments, with the distances being 9.2 m, 6.3 m, 3.5 m, and 3.3 m, respectively. As more sections are mined, the stress concentration zone shifts further into the upper left rock mass, with the peak stress concentration moving increasingly closer to the coal wall.

### The stress transfer characteristics of the surrounding rock

**Fig. 16:**
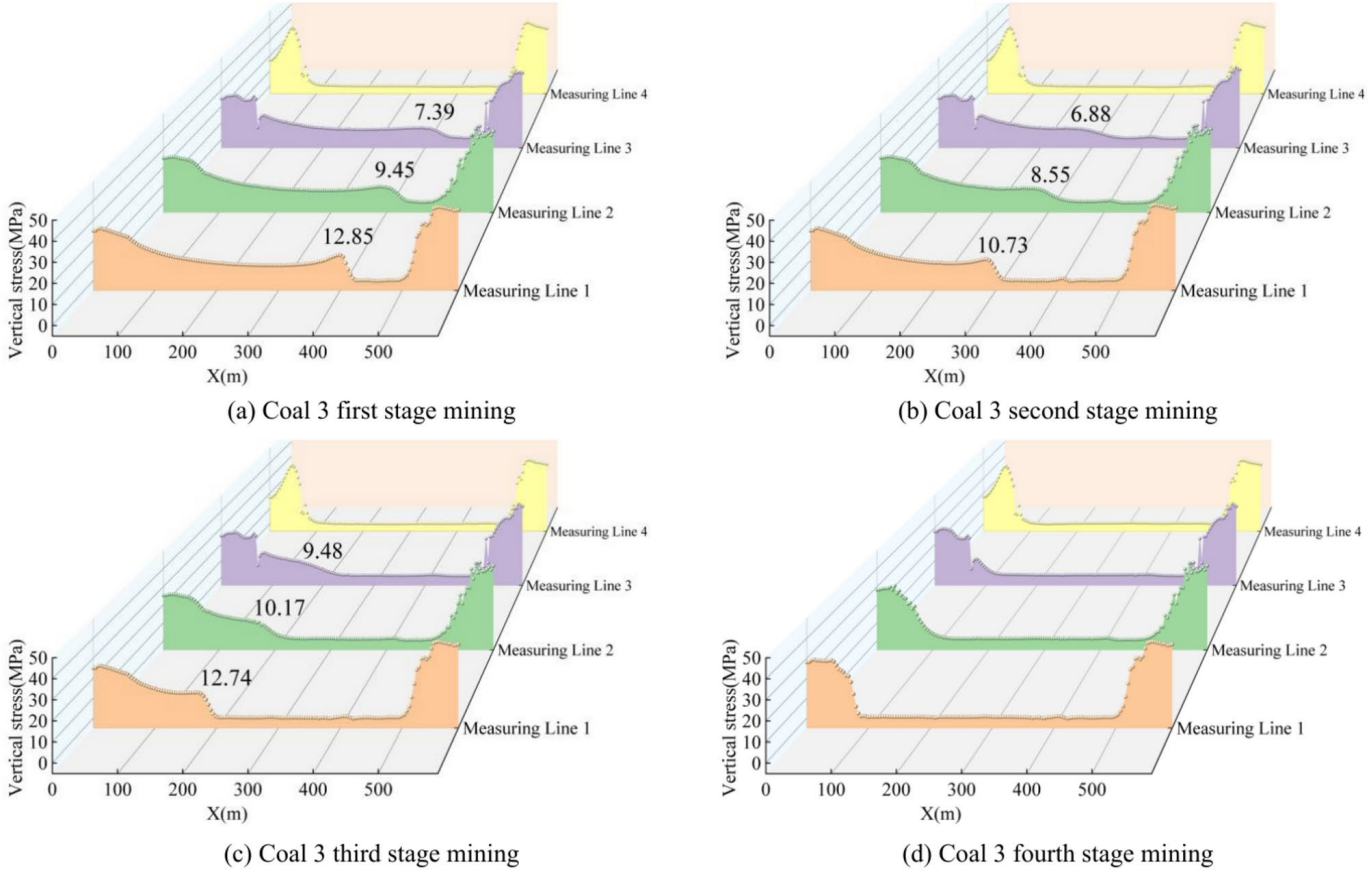
Features of stress transfer evolution.

From the above analysis, it can be seen that as inclined mining advances, the peak vertical stress in front of the advancing coal wall exhibits a trend of being lower in the middle and higher at both ends. The peak stress concentration on the advancing side moves increasingly closer to the coal wall. After the mining of the goafs in coal seams 1 and 3, vertical stress was measured and recorded using positioning measurement lines along the roof and floor of the coal seams. These lines were placed sequentially on the roof of coal seam 3, the floor of coal seams 2^−3^, the roof of coal seams 2^−3^, and the floor of coal seams 1. Figure 16 illustrates the stress transfer characteristics of the surrounding rock after mining of coal 1 and each section of coal 3.

After the mining of the upper coal seam 1, the vertical stress measured along each line showed a pattern of lower stress in the middle and higher stress at both ends. The stress peak in front of the coal wall on the roof of coal seam 3, after the first section of mining, was 12.85 MPa, with a pressure relief zone forming in the mining area. After the second section was mined, the stress peak in front of the coal wall was 10.73 MPa. After the third section, the stress peak in front of the coal wall was 12.74 MPa, and the pressure relief zone further expanded. After the fourth section, the stress peak in front of the coal wall overlapped with the stress concentration zone from the mining of coal seam 1, and the pressure relief zone reached its maximum extent.

For the measurement line on the floor between coal seams 2 and 3, the stress peaks in front of the coal wall after each section of mining were 9.45 MPa, 8.55 MPa, and 10.17 MPa, respectively, which were 26.85%, 20.78%, and 19.94% lower than the stress peaks measured on the roof of coal seam 3 at the same stages, with an average reduction of 22.52%. For the measurement line on the roof between coal seams 2 and 3, the stress peaks in front of the coal wall after the first and second sections of mining were only 7.39 MPa and 6.88 MPa, respectively, which were 42.50% and 35.88% lower than the peaks measured on the roof of coal seam 3 for the same sections. The stress measured along these lines was significantly reduced, indicating that stress was fully released and transferred. Vertically, the farther the distance from the mined coal seam, the lower the stress in the rock strata.

As the working face advances, the stress peak in front of the coal wall suddenly increases and then rapidly drops, a process accompanied by rock collapse and damage. This area is characterized by significant stress variation. Unlike the roof of coal seam 3, the floor and roof between coal seams 2 and 3 did not show a significant drop in stress after reaching the peak, indicating that the extent of rock collapse and damage was smaller. The upward mining of coal seams 2 and 3 has a feasible distance, and as the working face advances, the stress in the overlying rock exhibits a transfer and transmission evolution pattern.

## Engineering applications

**Fig. 17:**
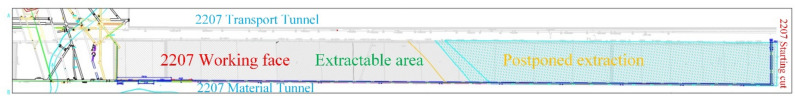
The tunnel layout plan of the 2207 working face.

Based on the feasibility analysis of upward mining, Xin’an Coal Mine is currently arranging the working face layout and tunnel excavation for coal seam 2. The 2207 working face is located in the southern wing of the near-horizontal mining area. It is the first working face for mining coal seam 2 in this near-horizontal area, with a length of approximately 1920 m. The total excavation work for the 2207 working face is designed to be 4508 m, which includes 2155 m for the 2207 transport tunnel, 2170 m for the 2207 material tunnel, and 183 m for the 2207 starting cut. Xin’an Coal Mine is currently conducting excavation work for the 2207 material tunnel. The 2207 material tunnel is being excavated from the 2207 material transport inclined shaft, exposing coal seam 2 and then advancing along the floor of the coal seam, with a total excavation length of 1989 m. The layout of the 2207 working face tunnels is shown in Fig. 17.

## Conclusion

1. Based on the “three-zone” discrimination method and the development pattern of the water-conducting fracture zone, the caving zone height of the overlying rock layers after the mining of coal seam 3 ranges from 6.86 to 11.26 m, while the fracture zone height ranges from 30.1 to 41.3 m. Coal seam 2^−3^ lie within the fracture zone after coal Seam 3 mining. Combined with the lower coal seam mining stress distribution to analyze the working face damage range, a formula for the depth of rock mass failure above the roof in an upward mining working face was derived. The analysis shows that the damage range at the edge of the working face under a plane stress condition is larger than that under a plane strain condition. The maximum failure height of the roof after the extraction of coal seam 3 is 10.04 m, which does not affect coal seam 2^−3^, indicating the feasibility of upward mining for coal seams 2^−3^.
2. Through physical similarity simulation experiments, it was observed that the caving zone height of coal seam 3 is approximately 9.3 m, which is 3.1 times the mining height. There is no significant damage to the roof and floor of coal seams 2^−3^, and the overall structure remains in a continuous layered state without any stepped displacement. During coal seam 4 mining, the caving zone height of the overlying strata is 12.6 m, with the fracture zone extending into the mined-out area of coal seam 3. The caving zone in coal seam 3 is minimally affected, and the overall continuous layered structure of coal seams 2^−3^ remains intact.
3. After the initial mining activity, the vertical stress in the working face exhibits regional characteristics. The stress distribution within the mining area, along the dip direction of the coal seam, can be divided into the original rock stress zone, the stress increase zone, the decompression zone, and the stress concentration zone. As coal seam 3 is mined upward, the stress concentration in front of the cutting entry continues to increase, with the peak stress concentration moving closer to the coal wall. The stress evolution characteristics of the roof and floor of coal seams 2^−3^ are significantly different from those of the roof of coal seam 3. After the peak stress occurs in the roof and floor of coal seams 2^−3^, there is no significant stress drop, and the degree of rock mass caving and failure is minimal, indicating that upward mining of coal seams 2^−3^ is feasible.

## Data Availability

All data generated or analyzed during this study are included in this published article.
